# The Management of an Abortion

**Published:** 1882-09

**Authors:** Matthew D. Mann

**Affiliations:** Professor of Obstetrics and Gynecology in University of Buffalo


BUFFALO

### Medical and Surgical Journal.

Vol. XXII. SEPTEMBER, 1882. No. 2.

### Original Communications.

### The Management of an Abortion.

By Matthew D. Mann, A. M., M. D.,

Professor of Obstetrics and Gynecology in University of Buffalo.

Scarcely any accident to which the pregnant woman is liable, is so likely to leave unpleasant consequences in its train, as an abortion. Occurring in the earlier months of gestation, when the union between the ovum and the uterus is very close, and before the uterine expulsive powers are well developed, unless art intervene, it is very apt to produce effects, which, although they may not be immediately fatal, are far reaching for evil.

The reason for this is that an abortion is an *abnormality* from the beginning. It is a product of civilization. Women in a state of health and nature never abort, save from violence; so that when the accident happens in our over-civilized poorly developed females, nature is taxed beyond her resources, and unless helped by art, is often entirely unable to complete the process which has been begun. In other words, nature never meant such a thing to happen and is consequently but imperfectly prepared to deal with it.

We are very often forced, therefore, as obstetricians, to take a prominent and active part in the management of the affair, and the danger is not of doing "meddlesome midwifery," but rather of trusting to nature a task for which she is often entirely incompetent.

The management of an abortion varies with the age of the ovum. In the first and second months very little is to be done. There is no placenta formed at that early period, and when the ovum comes away it generally does so entire, leaving nothing more than some few shreds of membrane which are not likely to do any harm. If we put our patient in bed until it is all over, and keep her there for a few days, we have done about all that is required in the way of treatment.

If the hemorrhage should happen to be excessive before the ovum comes away, a tampon may be applied, and on its removal the ovum will generally be found in the vagina. If there be a flow of any amount afterwards, it may be treated by a few applications of strong tincture of iodine to the inside of the uterus, or, if necessary, by removing with the curette any shreds of membrane which may remain.

The time when abortions are most apt to occur, is at the third menstrual epoch, after the last flow. At this time the placenta is beginning to be quite well formed, but the proportionate amount of maternal tissue involved in its structure, is much greater than when it is fully developed. This close union between the maternal tissues and the ovum is what causes the trouble and danger at this time. After the fifth month this danger no longer exists. Then the placenta is easily separated from the uterus and ordinarily gives no trouble.

Of abortions which occur in the third and fourth months, there are two classes which differ very much from one another in their results and in their demands for treatment. The first class includes the cases where the ovum is entire, and the second class comprises the cases where the sac ruptures and the fœtus escapes with the amniotic fluid.

*1st—When the sac is entire.* This is the rule in the first two months and may occur at any period of utero-gestation, particularly in those cases where the fœtus has been long dead. But, at the time we are speaking of—in the third and fourth month—it is the exception. If the patient be seen in the earlier stages, before the separation of the ovum from the membranes has advanced very far, then all our energies must be directed towards stopping the process and thus preventing the abortion. To this end the patient should be kept absolutely quiet in bed, opium given to accomplish this, if necessary, and full doses of the fluid extract of viburnum prunifolium administered every hour or two. Although my experience with this last drug has not been very extensive, I have considerable faith in it and can safely recommend it for further trial. It never produces any unpleasant symptoms as far as I know.

If these means do not have the desired effect, then we have little to do but to wait until the ovum is expelled. The tampon is scarcely ever necessary, as the unruptured ovum acts itself as a tampon, being forced into the cervical canal and held tightly there, owing to the undeveloped and undilated condition of the tissues surrounding it.

If the progress of the case is slow and the practitioner should be forced to leave the woman for a considerable length of time, as frequently occurs in the country, then the placing of a tampon as a precautionary measure is good practice; for such a case, although everything seems to be doing well, may be changed into a case of the second class at any moment by the bursting of the sac, and in such an event the presence of the tampon might save the woman's life.

It is needless to say that any measures, such as a violent examination or the passage of an instrument, which might jeopardize the integrity of the fœtal sac, should be carefully avoided. The woman's safety depends largely in the sac's remaining whole. After the ovum is expelled, the hemorrhage usually ceases and the uterus quickly returns to its natural size and position. To advance this process, the woman should be kept in bed and treated exactly as after labor at term. A neglect of this rule very often leads to uterine displacements and subinvolution.

*2d—When the ovum ruptures at an early stage* of the abortion, the hemorrhage is generally severe. The maternal tissues which are intimately blended with the fœtal in the formation of the placenta, are very vascular, and the sac which in the first class tampons the cervix and by its direct pressure on the blood-vessels tends to prevent hemorrhage, is now wanting. There is, therefore, nothing to stop the flow and it continues sometimes until the woman's life is endangered. The diagnosis of rupture of the sac can be made by the amount of flow.

Suppose we are called to such a case; we find that the woman has been bleeding severely for some time. We make an examination and find the uterus enlarged and the cervix dilated, what are the indications? To stop the hemorrhage as quickly as possible. To do this we must empty the uterus. The finger must be passed to the fundus and the remains of the ovum quickly swept out. The uterus will then contract and the flow be immediately arrested.

But suppose that the cervix is not dilated, what shall we do? In the presence of such a case we are met with a variety of recommendations. One says, give ergot and lead, put cold clothes over her vulva and wait. Another advises to tampon the vagina and give ergot, hoping thus to stop the hemorrhage and get the membrane and placenta out. Another tells us to dilate the cervix at once, with the woman under ether, and remove all that we can find in the uterus with the forceps or curette.

That the hemorrhage should be stopped, all will agree, and for this purpose there is nothing more effectual than a tampon, well applied. It gives us time to revive the woman with stimulants, to get assistance if we need it, and to make all necessary preparations.

After the tampon has been in place from 12 to 15 hours, it must be removed, and usually we will find the placenta free in the vagina. But, imagine, that on removing the tampon, we find the cervix still undilated and the flow as severe as ever. In that case there is no use waiting any longer for nature, but we must take matters into our own hands. Further use of the tampon alone is not likely to do any good. Moreover, it is painful and may become dangerous. After having washed out the vagina with an antiseptic solution, a Sims speculum should be used, and a large tupelo tent introduced into the cervix, care being taken to get it above the internal os. The vagina may then be lightly tamponed, enough to keep the tent in place and the woman left. If the cervix is too small to admit the introduction of a good sized tent, then the steel dilators may be first introduced and the cervix carefully stretched until the tent, or tents —for several small ones dilate better and faster than one large one—can be easily placed in position. I much prefer the tupelo tent to sponge or laminaria, as being less dangerous. After 12 hours the tampon and tents may be taken away and the finger passed into the uterus and its contents removed. The embryo is scarcely ever there, as it escapes with the water and is lost among the first clots.

To pass the finger into the uterus, the woman must be put across the bed, with the hips elevated. If the abdominal walls are relaxed, the uterus may be forcibly pressed down with one hand placed over the abdomen, while the index finger of the other is firmly and slowly pressed to the fundus. If the woman is very restless and hard to control, and the uterus tender and painful, it is much better to give ether before attempting these manipulations. If the abdomen is very resisting or fat, ether will help relax it, or the uterus may be seized by a double tenaculum and pulled down and held firmly with one hand, while the other works inside. In this way the whole of the placenta may, as a rule, be easily removed without danger to the woman.

After the placenta has been removed, all bleeding usually ceases. Sometimes, however, it persists and becomes formidable. Under such circumstances a hypodermic injection of ergot may be given; or, what is better, because more easily procured, an injection of hot water (120°) carried directly into the womb. In fact, I make it a routine practice to wash out the uterus with a hot carbolized injection in every case when my finger has been introduced into its cavity. If the hot water and ergot fail, it is a very bad case, other measures must be the same as those usually taken in post partum hemorrhage. The finger is better than any instrument, unless the placenta is merely extangled in the cervix. It is softer and more under control and less liable to do harm than the ordinary placental forceps.

Let us now suppose another case. If, after the embryo is expelled, the uterus contracts and the hemorrhage stops, either before or after the use of the tampon, what is to be done? Here again authorities differ. But, if we adopt the plan of the more conservative of our advisers and let things alone, what may be the course of events?

The late Prof. Spiegelberg has well summed up the possible results of what is well called an incomplete abortion.

1st. Gradual elimination with frequent hemorrhages.

2d. In exceptional cases, entire cessation of hemorrhage, and after a longer or shorter period, perhaps months, strong uterine contractions, severe hemorrhage and expulsion of the placenta.

3. More often, decomposition of the placenta. The woman is then exposed to septicæmia. Continuous fever is the result which may cease with the expulsion little by little of the decomposing mass; more commonly peri-uterine inflammation sets in and may leave the woman an invalid for years.

4. Rarely, the formation of a fibrinous polypus around the placenta.

5. Rarest of all, I may add, absorption of all the placental tissue. Dr. T. G. Thomas and others have reported such cases.

None of these contingencies, except the last, and that is so rare that Spiegelberg does not mention it, are at all pleasant to contemplate. The chances of the third result, the decomposition of the retained placenta, are so good that it should. it seems to me, be considered the rule and our practice regulated accordingly.

I never could feel satisfied about a patient after an abortion, no matter how well she might seem to be, if I knew that she had a placenta remaining in her womb. I should, therefore, advocate in every case the early removal of the remains of the abortion and the placing of the woman in the best possible condition for recovery. The method of removing the placenta in these cases differs in no respect from that already detailed. When we meet with cases which have been neglected and where a decomposing placenta is *in utero*, the one thing to do is to get it out without loss of time. Even if the woman has a high temperature and inflammation of the tissues around the uterus has begun, it is better to remove the cause and give the woman a chance, for as long as the placenta remains, so long is she in danger.

I have often met with cases where the placenta has remained for days in the cervical canal, the internal os as well as the external being tightly closed. Decomposition is the rule in these cases and, although the dangers of absorption are not so great, still its removal should not be delayed after its recovery. Dilatation of the external os and seizure of the mass with broad forceps easily accomplishes this.

I have said little of the use of disinfecting solutions in the various stages of treatment, because I suppose the necessity is well understood. The method of properly applying a tampon may be noticed, because it is so little appreciated and practiced. To properly tampon a vagina, common cotton wading (not absorbent) should be used. It should be made into balls, the size of a silver dollar, and then dipped into a solution of carbolic acid (ʒi to oj) and squeezed rather dry. There is no necessity of making a kite-tail affair, for this is cumbersome, takes time and is not so efficient. With a Sims tampon extractor, made on the principle of a gun-wormer, the cotton can be extracted quickly and without pain to the patient. Having everything ready, the patient should be placed in Sims position and a Sims speculum introduced; then with the dressing forceps the balls of cotton should be placed around the cervix, so as to encircle it and tightly compress it. Then one piece, previously soaked in a strong solution of alum, if you like, should be placed over the os and the remainder of the vagina tightly packed. Made in this way, a tampon will resist hemorrhage for sometime. My only excuse for giving these directions is that I consider the proper application of the tampon of the utmost importance.

The mere stuffing of a handkerchief or bandage into the vagina will do no good and offers very little hinderance to the flow of blood. If no speculum is at hand, the perineum may be lifted by two fingers, or a speculum may be improvised from a table-spoon.

It may be noticed that I have been very sparing in my recommendations of ergot. I know this is the sheet-anchor with many, but I must say that I do not believe in it. If given before the cervix is dilated in this class of cases, when the sac is not ruptured, by forcing the uterus to act too violently, it is liable to do just what we wish to avoid, viz.: cause the rupture of the sac. If used after the sac is ruptured and when the uterus is contracted around the placenta, instead of helping its expulsion, it only makes the uterus grasp it more firmly and, moreover, does little towards stopping the hemorrhage. The only instance when it may be used with advantage, is in the case already mentioned, when, after the uterus is thoroughly emptied, there is a continuance of the hemorrhage. In such a contingency a hypodermic injection of ergot may be of great help: but even here it is not so reliable as an intra-uterine injection of hot water.

